# Two Cases of Morel-Lavallée Lesion Which Resulted in a Wide Skin Necrosis from a Small Laceration

**DOI:** 10.1155/2020/5292937

**Published:** 2020-03-19

**Authors:** Tadahiro Nakajima, Kaoru Tada, Mika Nakada, Masashi Matsuta, Hiroyuki Tsuchiya

**Affiliations:** Department of Orthopaedic Surgery, Graduate School of Medical Science, Kanazawa University, 13-1 Takaramachi, Kanazawa 920-1302, Japan

## Abstract

Morel-Lavallée lesion (MLL) is a degloving injury in soft tissues caused by shear force accompanying trauma. Even if it is a small lacrimal wound at the initial visit, there is a range of skin necrosis which is not suitable for it. As a cause of the injury, a shearing force was applied over a wide range, and penetrating blood vessel damage to the skin occurred, resulting in skin necrosis. Attention is required.

## 1. Introduction

**Figure 1:**
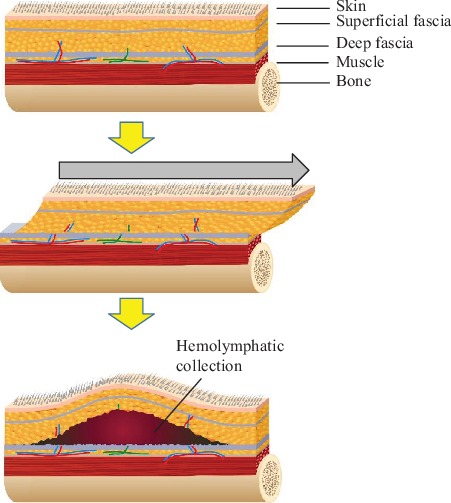
Illustration of Morel-Lavallée lesions. Tangential shearing force causes a closed soft tissue degloving injury, in which the skin and subcutaneous tissue are separated from the underlying fascia. This separation creates the space which can fill with blood and lymph.

Morel-Lavallée lesion (MLL) is defined as a closed, internal soft tissue degloving injury produced most commonly as a result of a strong, shearing, and tangential force [1]. This lesion is characterized by the development of a fluid collection between the subcutaneous soft tissue and the muscular fascia (Figure 1). Here, we present two cases of MLL that occurred in the distal thigh and lower leg which resulted in a wide skin necrosis.

## 2. Case Presentation

**Figure 2:**
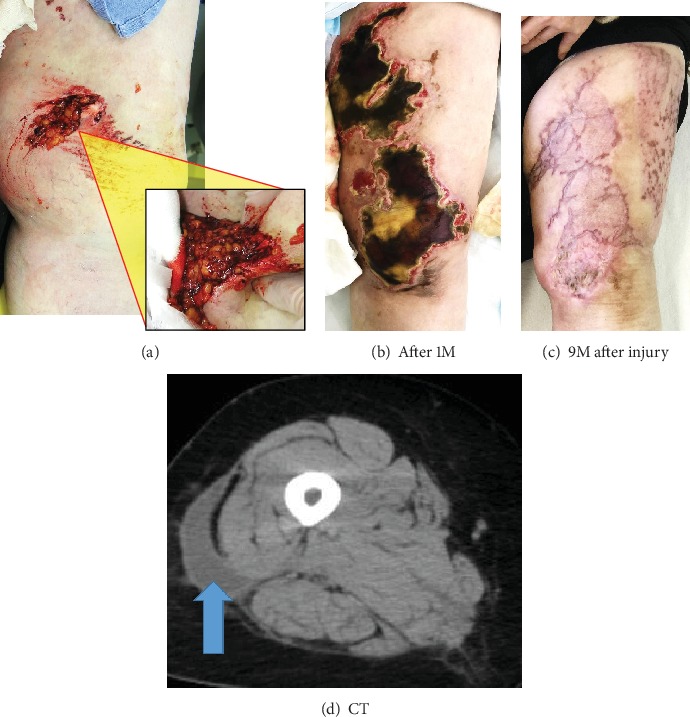
(a) Intraoperative photo. Fat masses came from a small size of wound. (b) Clinical photo. One month after the injury. Skin necrosis develops. (c) Clinical photo. Nine months after the injury. Skin grafts were performed, and the healing was complete. (d) CT scan performed three months after the injury. A small fluid collection between the subcutaneous soft tissue and the muscle fascia is indicated by an arrow.

Case 1 is a 66-year-old female, victim of a traffic accident. Her left thigh was caught in a car tire. She developed swelling and several contusions of her left thigh and sustained laceration of the medial side of her left knee. She underwent washout of her wound on that day. Intraoperatively, a small size of fat masses came out from the wound site (Figure 2(a)). After the washing, she underwent daily dressing changes and local wound care. One month after the injury, however, the wound failed to heal and skin necrosis developed on the anterior medial part of her left thigh (Figure 2(b)). She was admitted and underwent split-thickness skin grafting. After the skin graft, the healing was complete (Figure 2(c)). A computerized tomography (CT) scan performed three months after the injury demonstrated just a small fluid collection between the subcutaneous soft tissue and the muscle fascia (Figure 2(d)).

**Figure 3:**
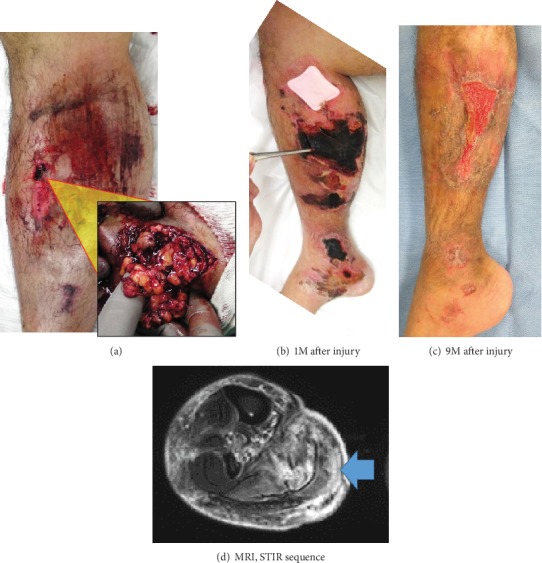
(a) Intraoperative photo. Fat masses came from a small size of wound. (b) Clinical photo. One month after the injury. Skin necrosis develops. (c) Clinical photo. Nine months after the injury. After the split-thickness skin graft, the healing was complete. (d) MRI performed one month after the injury. A small fluid collection between the subcutaneous soft tissue and the muscle fascia in indicated by an arrow.

Case 2 is a 26-year-old male who presented with swelling over the right lower leg. His legs were caught between the rollers during work. He sustained several contusions of the right lower leg and a laceration of the medial side of his right knee. He underwent irrigation and debridement on that day. Intraoperatively, a small size of fat masses came out from the wound site (Figure 3(a)). On the next day after the injury, compartment pressures of the right lower leg were over 40 mmHg. He underwent an emergent fasciotomy to leave the compartment open. Approximately three weeks after the injury, skin ulcer of the right lower leg advanced to necrosis (Figure 3(b)). He required serial debridements and negative pressure wound therapy (NPWT) dressings followed by split-thickness skin graft from his thigh. After the skin graft, the healing was complete (Figure 3(c)). Magnetic resonance imaging (MRI) performed one month after the injury showed just a small collection of fluid between the subcutaneous soft tissue and the muscle fascia (Figure 3(d)).

## 3. Discussion

MLL was first described by Morel-Lavallée in 1848 [2], and it is the sequela of a closed degloving injury involving separation of the skin and subcutaneous fat from the underlying fascia [3]. It has been well described as most commonly occurring in the greater trochanter, other common locations including elsewhere around the pelvis or thigh [4]. Less commonly occurring sites include the gluteal and lumbosacral regions, as well as the lower leg [5]. Moreover, Vanhegan et al. reviewed and identified a rare site of incidence as calf/lower leg (1.5%) [6]. The diagnosis of MLL is based on physical examination and imaging studies. Typical image finding of CT and MRI is a subcutaneous fluid collection [7]. In our two cases though, CT and MRI showed just a small fluid collection between the subcutaneous soft tissue and the underlying fascia. It is considered that fluid collection exuded subcutaneously was discharged from laceration. It is possible that once a shearing force is added and the penetrating branch is damaged, blood flow to the subcutaneous tissue does not recover and the skin falls into necrosis. That is why a wide skin necrosis which is incompatible with the small laceration at the initial examination occurred. It should be noted that in the cases of high-energy trauma, especially due to the shearing force, there may be cases where skin necrosis occurs extensively and healing is prolonged even though the wound is small.

## 4. Conclusion

A laceration that is thought to have been subjected to shear force may cause extensive skin necrosis afterwards and requires caution.
